# Diabetic Retinopathy and Clinical Parameters Favoring the Presence of Diabetic Nephropathy could Predict Renal Outcome in Patients with Diabetic Kidney Disease

**DOI:** 10.1038/s41598-017-01204-6

**Published:** 2017-04-21

**Authors:** Chi-Chih Hung, Hugo You-Hsien Lin, Daw-Yang Hwang, I-Ching Kuo, Yi-Wen Chiu, Lee-Moay Lim, Shang-Jyh Hwang, Hung-Chun Chen

**Affiliations:** 1 Division of Nephrology, Department of Internal Medicine, Kaohsiung Medical University Hospital, Kaohsiung Medical University, Kaohsiung, Taiwan; 2 Department of Internal Medicine, Kaohsiung Municipal Ta-Tung Hospital, Kaohsiung Medical University, Kaohsiung, Taiwan; 3 Faculty of Renal Care, College of Medicine, Kaohsiung Medical University, Kaohsiung, Taiwan

## Abstract

Diabetes duration, diabetic retinopathy (DR), and a diagnostic model have been proposed as clinical parameters favoring the presence of diabetic nephropathy (DN) in biopsied patients with diabetic kidney disease. DN, compared with non-diabetic renal disease, had poorer renal outcomes. We tested whether these clinical parameters favoring DN are associated with poorer renal outcomes in non-biopsied patients. In this study, 1330 patients with type 2 diabetes and chronic kidney disease stages 1–4 were included and divided according to diabetes mellitus (DM) duration >8 years, DR, or a diagnostic model for DN. These clinical parameters favoring DN were found in 62–77% of patients and associated with higher levels of proteinuria. In a Cox survival analysis, DR and the diagnostic model favoring DN were associated with an increased risk for end-stage renal disease with adjusted hazard ratios of 1.69 (95% CI: 1.16–2.45, *P* = 0.006) and 1.66 (95% CI: 1.05–2.61, *P* = 0.029), respectively. DR was associated with an increased risk for rapid renal disease progression. DM >8 years was not associated with renal outcome. Propensity score-matched analyses also showed similar results. In conclusion, DR and the diagnostic model favoring DN were associated with poorer renal outcomes.

## Introduction

The incidence and prevalence of type 2 diabetes mellitus (referred to as diabetes hereafter) are increasing worldwide^1^. Diabetes is the leading cause of chronic kidney disease (CKD) in many countries, and approximately 40% of patients subsequently develop diabetic kidney disease (DKD)^2^. DKD is the leading cause of end-stage renal disease (ESRD) in many developed countries^3^. The classic presentation of DKD is diabetic nephropathy (DN) characterized by albuminuria and nodular glomerulosclerosis. However, non-diabetic renal disease (NDRD) can present in biopsied patients with DKD, with immunoglobulin A (IgA) nephropathy and focal segmental glomerulosclerosis (FSGS) as the leading causes^4, 5^. In this study, we apply the term “DN” or “NDRD” to biopsy-proven cases and “DKD” to non-biopsied cases. Because NDRD is treatable and has a more favorable prognosis than that of DN^6–8^, renal biopsy is indicated in patients suspected to have NDRD.

Clinical differentiation between DN and NDRD is difficult. Several parameters, especially longer diabetes duration, diabetic retinopathy (DR), and absence of hematuria have been proposed as predictors for DN in recent meta-analyses^9, 10^. However, the pooled positive predictive value (PPV) for DN was measured to be only 0.72 in a meta-analysis of its most accurate predictor, DR^9^. A diagnostic model developed at a single center in China which included diabetes duration, systolic blood pressure, HbA1c, hematuria, hemoglobin and diabetic retinopathy as parameters, showed a PPV of 0.89 for DN^11^. These biopsy studies included specially selected patients with DKD, who usually presented with heavy proteinuria and were young^9, 10, 12^. These clinical parameters might not be applicable to non-selected patients with DKD. Thus, we propose an approach to test these parameters: if these predictive parameters can differentiate between DN and NDRD in patients with DKD, the DN patients favored by these parameters should have less favorable renal outcomes. However, in a study to investigate the prognostic value of retinopathy in assessing the risk of developing ESRD, cardiovascular morbidity or death among patients from the Trial to Reduce Cardiovascular Events with Aranesp Therapy (TREAT) study, DR was common but not independently associated with ESRD or other endpoints^13^. The predictive value of these parameters in DKD, especially DR and diabetes duration, is still controversial. We thus hypothesized that longer diabetes duration, DR and the diagnostic model favoring DN are associated with less favorable renal outcomes, cardiovascular events and all-cause mortality in non-biopsied patients with DKD.

## Results

### Baseline characteristics of patients with diabetic kidney disease by clinical parameters favoring diabetic nephropathy

The baseline characteristics of 1330 diabetic patients with CKD stages 1–4 are shown in Table 1. The mean age was 64.2 ± 12.7 years and the estimated glomerular filtration rate (eGFR) was 33.7 (24.2–46.3) mL/min/1.73 m^2^, with a urine protein-to-creatinine ratio (UPCR) of 928 (299–2536) mg/g and a glycated hemoglobin (HbA1c) level of 7.6% ± 1.8%. Of the patients, 67.1% had hypertension, 27.4% had cardiovascular (CV) disease, and 22.8% of the patients were treated with insulin. In 98 patients who underwent renal biopsy, 54 of them were diagnosed as DN. In multivariate logistic regression, we found that DR, absence of hematuria, low eGFR, and high HbA1c were associated with DN (Supplemental Table 1).

**Table 1:** Characteristics and outcomes of patients with diabetic kidney disease by clinical parameters favoring diabetic nephropathy.

Variable	All	DM > 8 years			Diabetic retinopathy			Diagnostic model		
		−	+	*P*-value	−	+	*P*-value	−	+	*P*-value
No. of patients	1330	300	1030	—	498	832		470	860	<0.05
Percentage in all patients		22.6%	77.4%		37.4%	62.6%		35.3%	64.7%	
**Demographics and comorbidity**										
Age (yr)	64.2 ± 12.7	62.9 ± 13.9	64.6 ± 12.3	0.059	64.4 ± 14.0	64.0 ± 11.8	0.604	63.1 ± 13.7	64.8 ± 12.0	0.019
Sex (female)	501 (37.7%)	109 (36.3%)	392 (38.1%)	0.587	183 (36.7%)	318 (38.2%)	0.591	127 (27.0%)	374 (43.5%)	<0.001
Cardiovascular disease	364 (27.4%)	39 (13.0%)	325 (31.6%)	<0.001	120 (24.1%)	244 (29.3%)	0.038	107 (22.8%)	257 (29.9%)	0.005
Ischemic heart disease	209 (15.7%)	21 (7.0%)	188 (18.3%)	<0.001	81 (16.3%)	128 (15.4%)	0.669	64 (13.6%)	145 (16.9%)	0.120
Congestive heart disease	147 (11.1%)	14 (4.7%)	133 (12.9%)	<0.001	50 (10.0%)	97 (11.7%)	0.362	39 (8.3%)	108 (12.6%)	0.018
Cerebrovascular disease	263 (19.8%)	30 (10.0%)	233 (22.6%)	<0.001	86 (17.3%)	177 (21.3%)	0.076	79 (16.8%)	184 (21.4%)	0.045
Hyperuricemia	200 (15.0%)	41 (13.7%)	159 (15.4%)	0.45	115 (23.1%)	85 (10.2%)	<0.001	101 (21.5%)	99 (11.5%)	<0.001
Hypertension	893 (67.1%)	98 (32.7%)	795 (77.2%)	<0.001	339 (68.1%)	554 (66.6%)	0.577	296 (63.0%)	597 (69.4%)	0.017
Smoker	184 (13.8%)	43 (14.3%)	141 (13.7%)	0.329	68 (13.7%)	116 (13.9%)	0.873	75 (16.0%)	109 (12.7%)	0.046
MBP (mmHg)	99.7 ± 13.5	99.5 ± 12.9	99.8 ± 13.7	0.745	99.2 ± 13.5	100.0 ± 13.6	0.34	97.4 ± 12.5	100.9 ± 13.9	<0.001
BMI (Kg/m^2^)	25.5 ± 4.1	25.4 ± 4.2	25.5 ± 4.0	0.788	25.3 ± 4.3	25.6 ± 3.9	0.188	25.8 ± 4.2	25.3 ± 4.0	0.063
**Diabetes status**										
DM > 8 years	1030 (77.4%)	0 (0%)	1030 (100%)	<0.001	332 (66.7%)	698 (83.9%)	<0.001	299 (63.6%)	731 (85.0%)	<0.001
DM retinopathy	832 (62.6%)	134 (44.7%)	698 (67.8%)	<0.001	0 (0%)	832 (100%)	<0.001	91 (19.4%)	741 (86.2%)	<0.001
DM neuropathy	314 (23.6%)	4 (1.3%)	310 (30.1%)	<0.001	65 (13.1%)	249 (29.9%)	<0.001	61 (13.0%)	253 (29.4%)	<0.001
HbA1c (%)	7.6 ± 1.8	7.8 ± 1.3	7.5 ± 1.9	0.007	6.8 ± 1.4	8.1 ± 1.8	<0.001	6.9 ± 1.5	8.0 ± 1.9	<0.001
**Kidney disease status**										
eGFR (ml/min/1.73 m^2^)	33.7 (24.2–46.3)	37.9 (26.7–53.4)	32.1 (23.5–44.4)	<0.001	35.3 (24.6–50.0)	32.5 (23.8–44.6)	0.003	41.0 (29.5–55.2)	30.0 (22.3–41.3)	<0.001
UPCR (mg/g)	928 (299–2536)	726 (251–2169)	1009 (324–2685)	0.002	588 (227–1585)	1259 (396–3189)	<0.001	434 (170–1277)	1358 (465–3282)	<0.001
Hematuria	172 (12.9%)	43 (14.3%)	129 (12.5%)	0.411	70 (14.1%)	102 (12.3%)	0.345	101 (21.5%)	71 (8.3%)	<0.001

DM: diabetes mellitus, MBP: mean blood pressure, BMI: body mass index, eGFR: estimated glomerular filtration rate, UPCR: urine protein to creatinine ratio, HbA1c: glycated hemoglobin.

Data are presented as the mean ± standard error, median (interquartile range), or count (percentage).

We divided our patients by three clinical parameters favoring DN (diabetes mellitus [DM] > 8 years, DR, and the positive diagnostic model) according to our data and the literature^4, 9, 10^. The proportions of DM > 8 years, DR, and the positive diagnostic model were 1030 (77.4%), 832 (62.6%), and 860 (64.7%), respectively (*P* < 0.05). Patients with DM > 8 years, DR, or the positive diagnostic model exhibited a higher prevalence of cardiovascular disease, lower eGFR, higher UPCR (Table 1), lower hemoglobin and albumin level, and higher phosphate level and a higher prevalence of insulin and angiotensin-converting enzyme inhibitor/angiotensin II receptor blocker treatment (Table 2), compared with patients without any of these three parameters (all *P* < 0.05). However, body mass index (BMI), cholesterol and C-reactive protein (CRP) were not different between groups. Patients with DM > 8 years had higher uric acid; patients with DR and the positive diagnostic model possessed higher HbA1c; and patients with the positive diagnostic model exhibited higher mean blood pressure and a higher prevalence of hematuria and female (all *P* < 0.05) (Table 1).

**Table 2:** Characteristics and outcomes of patients with diabetic kidney disease by clinical parameters favoring diabetic nephropathy.

Variable		DM > 8 years			Diabetic Retinopathy			Diagnostic Model		
	All	−	+	*P*-value	−	+	*P*-value	−	+	*P*-value
**Laboratory data**										
Hemoglobin (g/dl)	11.9 ± 2.2	12.3 ± 2.1	11.8 ± 2.2	<0.001	12.4 ± 2.2	11.6 ± 2.1	<0.001	13.3 ± 2.0	11.1 ± 1.9	<0.001
Albumin (g/dl)	3.8 ± 0.6	3.9 ± 0.6	3.8 ± 0.6	0.005	3.9 ± 0.6	3.8 ± 0.6	0.001	4.0 ± 0.6	3.7 ± 0.6	<0.001
Blood glucose (mg/dl)	137 ± 56	133 ± 52	138 ± 57	0.197	115 ± 38	150 ± 61	<0.001	119 ± 45	147 ± 59	<0.001
Total cholesterol (mg/dl)	194 (165–225)	198 (170–226)	193 (164–225)	0.092	192 (169–225)	194 (163–225)	0.572	194 (169–224)	194 (163–226)	0.781
Triglyceride (mg/dl)	139 (101–206)	147 (109–215)	136 (100–199)	0.036	131 (95–193)	145 (105–211)	0.004	133 (100–197)	143 (102–210)	0.146
Sodium (mEq/l)	138.2 ± 3.6	138.8 ± 3.3	138.1 ± 3.6	0.001	138.9 ± 3.2	137.8 ± 3.7	<0.001	139.1 ± 3.1	137.7 ± 3.7	<0.001
Potassium (mEq/l)	4.3 ± 0.5	4.3 ± 0.5	4.3 ± 0.5	0.931	4.2 ± 0.5	4.3 ± 0.5	<0.001	4.2 ± 0.5	4.3 ± 0.5	<0.001
Phosphorus (mg/dl)	4.0 ± 0.9	3.8 ± 0.8	4.0 ± 0.9	0.002	3.8 ± 0.8	4.0 ± 0.9	<0.001	3.7 ± 0.8	4.1 ± 0.9	<0.001
Calcium (mg/dl)	9.3 ± 0.7	9.4 ± 0.7	9.2 ± 0.7	<0.001	9.3 ± 0.6	9.2 ± 0.7	0.024	9.4 ± 0.6	9.2 ± 0.7	<0.001
Uric acid (mg/dl)	7.6 ± 1.9	7.4 ± 2.0	7.7 ± 1.9	0.034	7.6 ± 1.9	7.6 ± 1.9	0.563	7.6 ± 2.0	7.6 ± 1.9	0.644
C-reactive protein (mg/l)	1.2 (0.4–5.8)	1.6 (0.3–8.8)	1.2 (0.4–5.5)	0.177	1.3 (0.3–5.9)	1.2 (0.4–5.7)	0.965	1.1 (0.3–5.2)	1.3 (0.4–6.1)	0.064
**Medications**										
ACEI/ARB	850 (63.9%)	162 (54.0%)	688 (66.8%)	<0.001	266 (53.4%)	584 (70.2%)	<0.001	257 (54.7%)	593 (69.0%)	<0.001
Other anti-HTN agent	303 (22.8%)	33 (11.0%)	270 (26.2%)	<0.001	96 (19.3%)	207 (24.9%)	0.018	74 (15.7%)	229 (26.6%)	<0.001
Insulin treatment	304 (22.8%)	42 (14.0%)	262 (25.4%)	0.006	20 (4.0%)	284 (34.2%)	<0.001	18 (3.8%)	286 (33.2%)	<0.001
**Outcomes**										
Follow-up days	1064 (665–1644)	1117 (619–1691)	1037 (674–1643)	0.701	1059 (616–1641)	1071 (700–1654)	0.425	1069 (616–1610)	1061 (684–1672)	0.320
eGFR slope (ml/min/1.73 m^2^/yr)	−2.4 (−6.8 to 0.4)	−1.4 (−5.7 to 1.3)	−2.7 (−7.2 to 0.1)	<0.001	−1.3 (−4.5 to 1.3)	−3.3 (−7.9 to −0.2)	<0.001	−0.9 (−3.8 to 1.6)	−3.5 (−8.0 to −0.6)	<0.001
Rapid renal progression	442 (33.8%)	79 (27.8%)	363 (35.4%)	0.016	115 (23.6%)	327 (39.8%)	<0.001	97 (21.2%)	345 (40.6%)	<0.001
ESRD	208 (15.7%)	24 (8.0%)	184 (17.9%)	<0.001	40 (8.0%)	168 (20.2%)	<0.001	24 (5.1%)	184 (21.4%)	<0.001
All-cause mortality	185 (13.9%)	43 (14.3%)	142 (13.8%)	0.810	55 (11.0%)	130 (15.6%)	0.020	45 (9.6%)	140 (16.3%)	0.001
Cardiovascular events	215 (16.2%)	31 (10.3%)	184 (17.9%)	<0.001	61 (12.2%)	154 (18.5%)	<0.001	44 (9.4%)	171 (19.9%)	<0.001

ACEI, angiotensin-converting enzyme inhibitor; ARB, angiotensin II receptor blocker; Anti-HTN, anti-hypertensive; ESRD: end-stage renal disease. Data are presented as the mean ± standard error, median (interquartile range), or count (percentage).

### Factors associated with DM > 8 years and DR in patients with diabetic kidney disease

In multivariate logistic regression analysis, CV disease was associated with a higher odds ratio (OR) for DM > 8 years, whereas the eGFR and CRP were associated with lower ORs for DM > 8 years (all *P* < 0.05) (Table 3). By contrast, the UPCR, HbA1c, and BMI were associated with higher ORs for DR, whereas male patients, hemoglobin, and cholesterol were associated with lower ORs for DR (all *P* < 0.05) (Table 3).

**Table 3:** Factors associated with DM > 8 years and diabetic retinopathy from patient without biopsy confirmed diabetic nephropathy.

	DM > 8 years			Diabetic Retinopathy		
	Odds ratio	95% CI	*P*-value	Odds ratio	95% CI	*P*-value
Age (yr)	1.002	0.991 to 1.013	0.736	0.999	0.989 to 1.010	0.900
Male vs. female	0.956	0.694 to 1.317	0.784	0.734	0.546 to 0.987	0.041
eGFR (ml/min/1.73 m^2^)	0.990	0.983 to 0.997	0.006	0.997	0.990 to 1.005	0.477
Log-transformed UPCR	1.234	0.927 to 1.642	0.149	1.743	1.327 to 2.290	0.000
Cardiovascular disease	2.685	1.850 to 3.896	0.000	1.321	0.986 to 1.769	0.062
MBP (mmHg)	1.002	0.992 to 1.013	0.667	0.998	0.989 to 1.008	0.758
HbA1c (%)	0.944	0.876 to 1.017	0.129	1.784	1.617 to 1.968	0.000
Smoker	1.081	0.729 to 1.603	0.698	0.981	0.676 to 1.423	0.918
Hemoglobin (g/dl)	0.993	0.914 to 1.080	0.875	0.829	0.766 to 0.898	0.000
Albumin (g/dl)	0.810	0.594 to 1.105	0.184	1.378	1.037 to 1.831	0.027
Log-transformed CRP	0.851	0.736 to 0.983	0.028	0.872	0.759 to 1.001	0.052
BMI (Kg/m2)	1.015	0.980 to 1.052	0.394	1.040	1.007 to 1.075	0.017
Log-transformed cholesterol	0.331	0.095 to 1.156	0.083	0.155	0.048 to 0.497	0.002
Phosphorus (mg/dl)	1.123	0.934 to 1.349	0.217	1.168	0.985 to 1.385	0.074
Uric acid (mg/dl)	1.011	0.937 to 1.090	0.779	0.981	0.914 to 1.052	0.590
Hematuria	0.783	0.527 to 1.163	0.226	0.724	0.497 to 1.056	0.094

DM: diabetes mellitus, MBP: mean blood pressure, BMI: body mass index, eGFR: estimated glomerular filtration rate, UPCR: urine protein to creatinine ratio, CRP: c-reactive protein, HbA1c: glycated hemoglobin.

### Associations between clinical parameters favoring diabetic nephropathy and renal outcomes in patients with diabetic kidney disease

**Figure 1:**
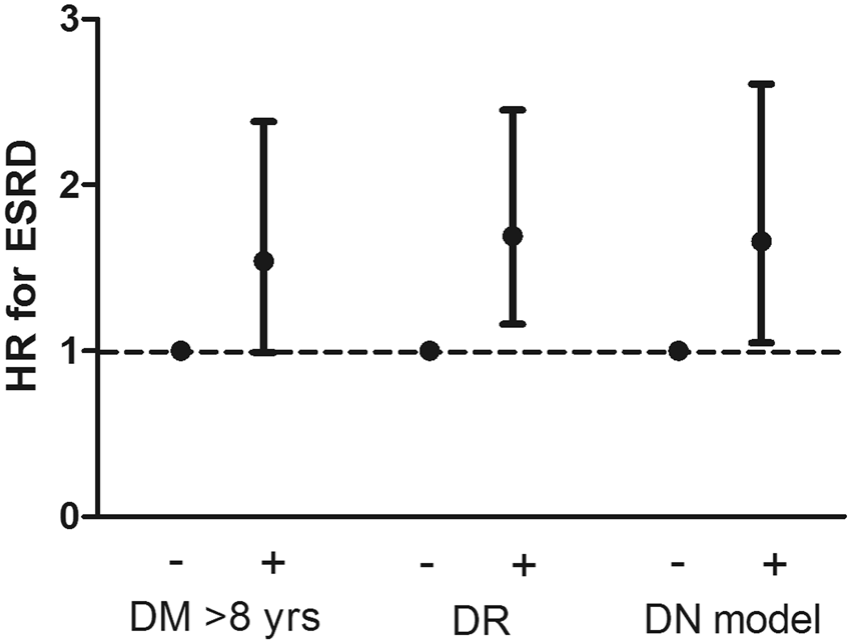
Association between clinical parameters favoring diabetic nephropathy and hazard ratios for end-stage renal disease(ESRD) in patients with diabetic kidney disease.

After a median follow-up period of 2.9 years, 24 (8.0%) and 184 (17.9%) patients with DM ≤ 8 years and DM > 8 years progressed to ESRD, respectively (Table 2). In the fully adjusted Cox proportional hazards model, DM > 8 years was associated with a trend of an increased risk for ESRD with a hazard ratio (HR) of 1.54 (95% confidence interval [CI]: 0.99–2.38, *P* = 0.055), compared with DM ≤ 8 years (Table 4) (Fig. 1). A total of 40 (8.0%) patients without and 168 (20.2%) patients with DR progressed to ESRD, respectively, and 24 (5.1%) patients without and 184 (21.4%) patients with the positive diagnostic model progressed to ESRD, respectively. Both DR and the positive diagnostic model were significantly associated with increased risks for ESRD with HRs of 1.69 (95% CI: 1.16–2.45, *P* = 0.006) and 1.66 (95% CI: 1.05–2.61, *P* = 0.029), respectively (Table 4) (Fig. 1). In the fully adjusted multivariate logistic regression, DR was significantly associated with an increased risk for rapid renal progression with an OR of 1.55 (95% CI: 1.5–2.09, *P* = 0.004) (Table 4).

**Table 4:** Associations between clinical parameters favoring diabetic nephropathy and clinical outcomes in patients with diabetic kidney disease.

	DM > 8 years			Diabetic retinopathy			Diagnostic model		
	−	+	*P*-value	−	+	*P*-value	−	+	*P*-value
***HR for ESRD***									
Unadjusted	1 (reference)	2.30 (1.50–3.52)	<0.001	1 (reference)	2.56 (1.81–3.61)	<0.001	1 (reference)	4.35 (2.84–6.66)	<0.001
Fully-adjusted	1 (reference)	1.54 (0.99–2.38)	0.055	1 (reference)	1.69 (1.16–2.45)	0.006	1 (reference)	1.66 (1.05–2.61)	0.029
***OR for rapid renal progression***									
Unadjusted	1 (reference)	1.43 (1.07–1.90)	<0.001	1 (reference)	2.14 (1.67–2.75)	<0.001	1 (reference)	2.54 (1.96–3.31)	<0.001
Fully-adjusted	1 (reference)	1.34 (0.96–1.87)	0.080	1 (reference)	1.55 (1.15–2.09)	0.004	1 (reference)	1.38 (0.96–1.98)	0.082
***HR for all-cause mortality***									
Unadjusted	1 (reference)	0.95 (0.68–1.34)	0.783	1 (reference)	1.38 (1.01–1.90)	0.044	1 (reference)	1.66 (1.18–2.32)	0.003
Fully-adjusted	1 (reference)	0.80 (0.57–1.15)	0.143	1 (reference)	1.02 (0.72–1.44)	0.921	1 (reference)	0.96 (0.65–1.40)	0.821
***HR for CV events***									
Unadjusted	1 (reference)	1.30 (0.98–1.73)	0.069	1 (reference)	1.54 (1.21–1.98)	0.001	1 (reference)	1.89 (1.45–2.46)	<0.001
Fully-adjusted	1 (reference)	0.94 (0.73–1.33)	0.547	1 (reference)	1.33 (1.01–1.74)	0.045	1 (reference)	1.10 (0.82–1.47)	0.521

Model adjusts for age, sex, eGFR, log-transformed UPCR, HbA1c, hypertension, cardiovascular disease, MBP, albumin, hemoglobin, BMI, log-transformed CRP, BMI, log-transformed cholesterol, phosphate and ACEI/ARB. *P < 0.0.

### Association between clinical parameters favoring diabetic nephropathy and clinical outcomes in patients with diabetic kidney disease

**Figure 2:**
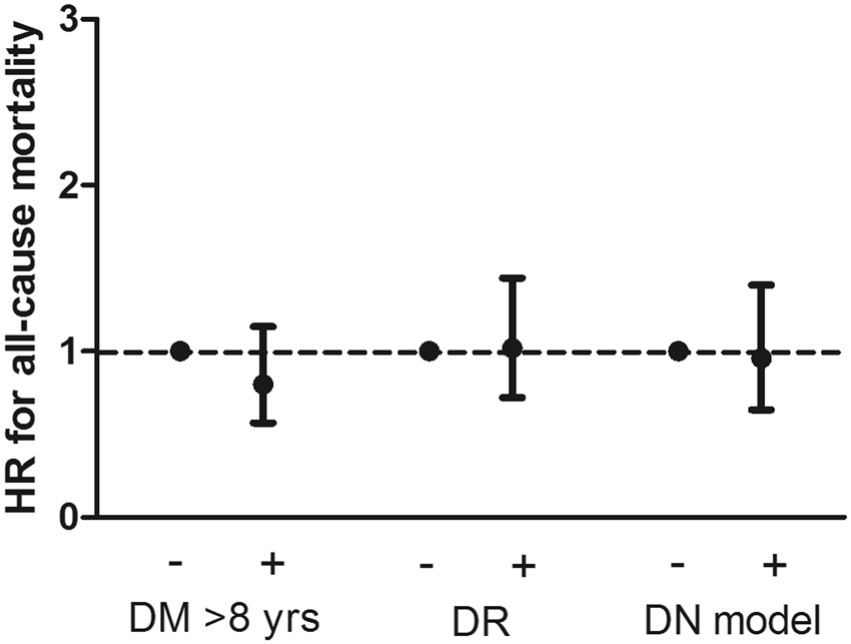
Association between clinical parameters favoring diabetic nephropathy and hazard ratios for all-caused mortality in patients with diabetic kidney disease.

**Figure 3:**
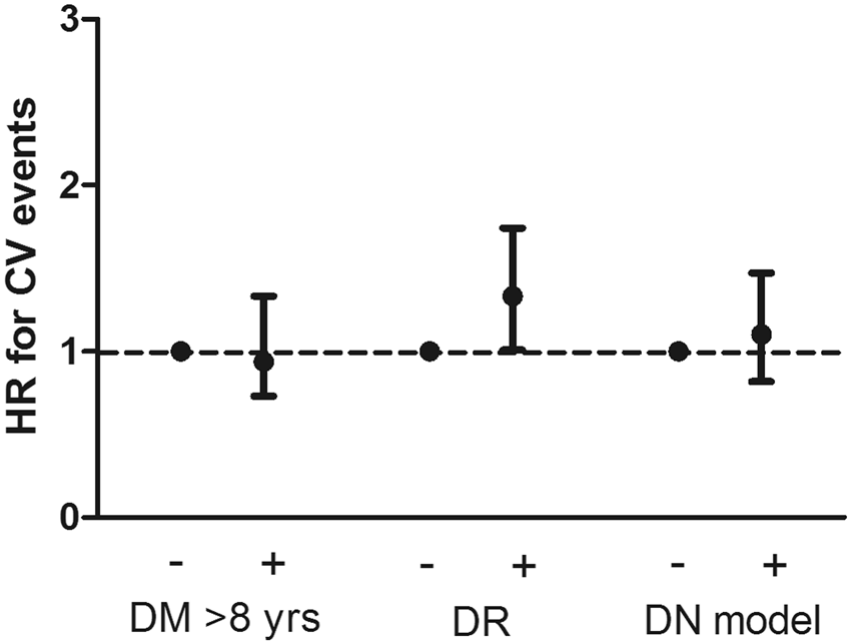
Association between clinical parameters favoring diabetic nephropathy and hazard ratios for cardiovascular (CV) events in patients with diabetic kidney disease.

In the fully adjusted Cox proportional hazards model, DM > 8 years, DR, and the positive diagnostic model were not associated with all-cause mortality (Table 4) (Fig. 2). A total of 31 (10.3%), 61 (12.2%), and 44 (9.4%) CV events occurred in the patients without DM > 8 years, DR, and the positive diagnostic model, respectively, and 184 (17.9%), 154 (18.5%), and 171 (19.9%) CV events occurred in the patients with DM > 8 years, DR, and the positive diagnostic model, respectively (Table 2). In the fully adjusted Cox proportional hazards model, DR was associated with a significantly increased risk for CV events with an HR of 1.33 (95% CI: 1.01–1.74, *P* = 0.045) (Table 4) (Fig. 3).

### Propensity score-matched analysis

Because these three parameters were associated with CKD stages and other factors, we performed propensity score matching according to all variables listed in supplement Table 2. There were 296 vs 296, 368 vs 368, 308 vs 308 patients in the analyses by DM > 8 years, DR and the diagnostic model, respectively. There was no differences in all these variables between the groups divided by these parameters. The results were similar in renal outcomes. Both DR and the positive diagnostic model were significantly associated with increased risks for ESRD with HRs of 1.80 (95% CI: 1.14–2.86, *P* = 0.012) and 1.89 (95% CI: 1.02–3.52, *P* = 0.045), respectively (supplementary Table 3). DR was significantly associated with an increased risk for rapid renal progression with an OR of 1.56 (95% CI: 1.07–2.26, *P* = 0.020). However, DR was not associated with an increased risk for CV events with an HR of 1.28 (95% CI: 0.85–1.92, *P* = 0.231).

## Discussion

Our study investigated whether DM > 8 years, DR, and the diagnostic model favoring DN are associated with clinical outcomes in patients with diabetic kidney disease. The presence of these clinical parameters, with a prevalence ranging from 62% to 77%, was associated with a higher incidence of CV disease, lower eGFR, and higher UPCR. We further revealed that DR and the diagnostic model favoring DN were significantly associated with an increased risk for ESRD. Diabetes duration was not associated with clinical outcomes.

DN is characterized by the development of albuminuria with renal disease progression. The typical pathological findings include glomerular basement membrane thickening, mesangial expansion, and nodular and global glomerulosclerosis^14^. NDRD is rarely comorbid with type 1 diabetes mellitus, particularly in patients with a history of diabetes >10 years^15^; however, reports of the prevalence of NDRD in patients diagnosed with type 2 diabetes mellitus have varied widely among studies, ranging from 17% to more than 50% according to different biopsy indications^16–18^. The most common cause of NDRD is IgA nephropathy in Asian countries and FSGS in Western countries^4, 19^. Various clinical parameters have been proposed as predictors for DN^10^ according to different biopsy policies and geographic areas^12^. A recent meta-analysis found that the presence of DR, longer diabetes duration, higher HbA1c, higher blood pressure (BP), and presence of hematuria could differentiate DN from NDRD; age, urine protein excretion, and serum creatinine could not^10^. Because DN had less favorable clinical outcomes than did NDRD^6–8^, we tested whether non-biopsied patients with clinical parameters favoring DN had poorer renal outcomes.

Researchers disagree on whether diabetes duration is a reliable predictor for DN. A meta-analysis found that it was reliable^10^, and a large original study by D’Agati *et al*. demonstrated that diabetes duration was the strongest predictor for DN in multivariate analysis, and that DM > 8 years was the optimal cutoff for predicting DN^4^. However, Parving *et al*. and two large original studies did not find a difference in diabetes duration between DN and NDRD^12, 20–22^. Because diabetes may have developed long before the diagnosis, the known diabetes duration has not been found to accurately predict the presence of DN^10, 23^. Additionally, in our cohort, more than half of the patients had a junior high school education level and might receive a delayed diagnosis of diabetes. These factors suggest that diabetes duration is not an accurate predictor for DN. Our result further demonstrated that diabetes duration is not a reliable prognostic factor for renal outcomes in patients with DKD.

DR is probably the most accurate single predictor for DN, but it is not perfect^9, 10^. Retinal microvascular abnormalities are correlated with glomerular lesions in biopsied patients with type 1 diabetes and associated with renal dysfunction in the general population with or without diabetes^24^. As mentioned previously, a higher incidence of NDRD is observed in cases of type 2 diabetes. Two meta-analyses demonstrated that DR could differentiate DN from NDRD with a pooled sensitivity of 0.65 and a pooled specificity of 0.75^9, 10^. Several original studies have also shown that DR is the most accurate predictor for DN in multivariate analysis^7, 8, 15^. Both proliferative and non-proliferative DR can be associated with DN^25^, with proliferative DR possibly being the more sensitive of the two^9^. However, DR has limitations as an indicator for DN. First, the literature derived from biopsy studies is biased by selection criteria that favor NDRD. Second, many patients with DR did not develop macroalbuminuria within 10 years of follow-up^26^. New biomarkers for differentiation between DN and NDRD deserve further study^27^. In addition to these limitations in differentiation, the prognostic value of DR for renal outcomes is controversial. DR has been associated with a faster decline in the eGFR among the general population and the elderly population with or without diabetes^24, 28^. Reports from the Action to Control Cardiovascular Risk in Diabetes (ACCORD) study in patients with early DKD and Reduction of Endpoints in NIDDM with the Angiotensin II Antagonist Losartin (RENAAL) study in patients with advanced DKD have found that DR is associated with renal composite endpoints (ESRD or the doubling of creatinine)^29, 30^. However, a recent report from the TREAT study did not find a significantly increased risk for ESRD in patients with advanced DKD and anemia^13^. Our result, demonstrated the association of DR with ESRD, is consistent with the report from the RENAAL study. The discrepancy between these studies of advanced DKD is probably attributable to a higher prevalence of DR and greater proteinuria in the RENAAL study and our study compared with the TREAT study. Future large studies such as DIACORE, a prospective cohort study of incident microvascular and macrovascular complications in 6000 patients with diabetes^31^, will be helpful for clarifying this relationship.

A single clinical parameter is insufficient to clearly differentiate DN from NDRD^32^. A differential diagnostic model composed of diabetes duration, blood pressure, HbA1c, hematuria, hemoglobin, and DR was proposed to predict DN^11^. The authors developed the model from a biopsy cohort with 178 patients and validated their results in another cohort of 55 patients with a sensitivity of 88.5%^11^. In our study, the prevalence of DN estimated according to DR was similar to that estimated according to this diagnostic model, and the prognostic value of DR for ESRD was similar to that of this diagnostic model (Table 4) (Fig. 1). Our study is the first to apply what we have found in the biopsied patients to non-biopsied patients with DKD. Larger studies are required to establish a more sophisticated diagnostic model for DN.

Hematuria is proposed as a predictive parameter for NDRD but may not be an effective exclusionary parameter for DN. A meta-analysis and several original studies have found that hematuria is a predictor for NDRD^10^. However, these studies exhibited heterogeneity, especially on the definition of hematuria. Our previous study has shown that hematuria according to ≥5–10 red blood cells per high power field (RBC/HPF), but not 2–5 RBC/HPF, was associated with NDRD^33^. This is supported by another report^11^. The prevalence of hematuria was approximately 15% in patients with DKD and ranged widely from 11% to 77% among biopsy studies^16–18^. The absence of hematuria alone is not a sufficient predictor for DN. Thus, we did not include hematuria as a predictive parameter in this study.

Our study findings have several limitations. First, we did not perform biopsies on the kidneys of randomly selected patients with DKD. The association of these clinical parameters with DN was evident only in biopsy studies. However, our purpose is to demonstrate that the evidence we learned from biopsy studies could be tested in non-biopsied patients, but not to substitute renal biopsy. Second, we proved the association between clinical parameters favoring DN and clinical outcomes, but not the direct association between DN and clinical outcomes. Extrapolating these results should be careful. Third, the sensitivity of clinical parameters and the diagnostic model for DN is high but nonetheless flawed. Some patients with DR might also have NDRD. Fourth, the severity of DR was not documented, and proliferative DR might be associated with a higher risk for ESRD. Fifth, diabetes duration was not an accurate predictor in our study. This is related to the low education level of our study population in whom signs of DM can’t be reported early. Sixth, there may be a selection bias involved in recruiting patients from the nephrology outpatient department. DKD patients form this recruitment might have higher percentage of NDRD.

## Conclusion

In our study, we tested whether clinical parameters favoring DN could predict clinical outcomes in patients with DKD. DR and the diagnostic model favoring DN were found in two thirds of patients with diabetes and CKD stages 1–4 and were associated with a higher proportion of CV disease, lower eGFR, and higher UPCR. DR and the diagnostic model favoring DN were significantly associated with an increased risk for ESRD. Further biomarker studies on the precise prediction of DN and clinical outcomes are warranted^27^.

## Methods

### Participants and measurements

A prospective observation study, the Integrated CKD Care Program Kaohsiung for Delaying Dialysis, was conducted at two hospitals affiliated with Kaohsiung Medical University in southern Taiwan from November 11, 2002 to May 31, 2009 with follow-up until May 31, 2010^34^. The study included patients who were not receiving renal replacement therapy and excluded patients with acute kidney injury defined as a greater than 50% decrease in the eGFR in 3 months. Among 3659 patients with CKD, we included those diagnosed with diabetes as defined by the World Health Organization^35^ who lacked significant ketonuria and had been receiving insulin treatment for at least 1 y after diagnosis. To observe renal outcomes, we excluded patients diagnosed with CKD stage 5; a final total of 1330 patients were eligible for this study. The study protocol was approved by the Institutional Review Board of Kaohsiung Medical University Hospital (KMUH-IRB-990198). Written informed consent was obtained from the patients and all clinical investigations were conducted according to the principles expressed in the Declaration of Helsinki.

CKD was defined as abnormalities of kidney structure or function, present for more than 3 months, with implications for health^36^. DKD was defined as the presence of both diabetes and CKD^37^. Diabetes duration was defined as the period between the time of diagnosis by a physician and the time of enrollment. DN, as stated in the introduction, referred to the definition by renal pathology^37^. DR was defined as the presence of background, preproliferative, and proliferative changes determined through fundoscopy or a digital fundus photography examination. Diabetic neuropathy was defined as generalized peripheral neuropathy determined through a nerve conduction velocity test. Microscopic hematuria was defined according to a rate ≥5–10 RBC/HPF. An automated cation-exchange high-performance liquid chromatography method was used to measure HbA1c. A six-parameter diagnostic model for DN including diabetes duration, blood pressure, HbA1c, hematuria, anemia and diabetes retinopathy was used. It was established and validated by Chen *et al*. as *P*_DN_ = exp (0.846 + 0.022 DM + 0.033 BP + 2.050 HbA1c − 2.664 Hu − 0.078 Hb + 2.942 DR)/(1 + exp [0.846 + 0.022 DM + 0.033 BP + 2.050 HbA1c − 2.664 Hu − 0.078 Hb + 2.942 DR])^11^. *P*_DN_ was the probability of DN diagnosis (*P*_DN_ ≥ 0.5 as DN). The units of the parameters are listed as follows: diabetes duration (mo); BP (mmHg); HbA1c (1 ≥ 7%, 0 < 7%); hematuria (1 = yes, 0 = no); Hb (g/L); and DR (1 = yes, 0 = no). Three parameters (DM > 8 years^4^, DR^9, 10^, and the diagnostic model^11^) were chosen as predictors for DN in this study. The absence of hematuria was not selected as a predictor for DN because our previous study had already addressed this question.

The baseline comorbidities of the patients, clinical data, and biochemical parameters were studied. The demographic features were recorded at the first visit and the medical history was recorded in a chart review. Hypertension was defined as systolic BP > 140 mmHg, diastolic BP > 90 mmHg, or the use of antihypertensive medication. Normotensive patients who took ACEI/ARB were not classified as hypertension. CV diseases were defined as a clinical diagnosis of heart failure, acute or chronic ischemic heart disease, or cerebrovascular disease. Laboratory data were also obtained at the baseline visit.

### Outcomes

Four outcomes were assessed: ESRD, rapid renal progression, all-cause mortality, and CV events. ESRD was defined as the initiation of hemodialysis, peritoneal dialysis, or renal transplantation. The initiation of ESRD was ascertained by reviewing patient charts and the catastrophic illness cards issued by Taiwan National Health Insurance. Kidney function was examined through the simplified Modification of Diet in Renal Disease (MDRD) study equation: eGFR mL/min/1.73 m^2^ = (186) × (serum creatinine^−1.154^) × (age^−0.203^) × 0.742 (if female) × 1.212 (if black). Rapid renal disease progression was defined as an eGFR slope <−5 mL/min/1.73 m^2^/y according to the Kidney Disease: Improving Global Outcomes (KDIGO) guideline. Survival status and cause of death were determined on the basis of death certificates, patient charts, and the National Death Index. CV events were ascertained by reviewing charts and defined according to hospitalization for acute coronary syndrome (Deyo’s modified Charlson score, ICD-9-CM: 410.x–412.x), acute cerebrovascular disease (430.x–438.x), congestive heart failure (428.x), or peripheral arterial occlusion disease (443.9, 441.x, 785.4, V43.4, procedure 38.48), or death by any of the aforementioned causes.

### Statistical analysis

The summarized statistical results of the baseline characteristics of the patients were expressed as counts and percentages for the categorical data. The means with standard deviations and medians with interquartile ranges were determined for continuous variables with approximately normal distributions. Competing risk Cox proportional hazards analysis was used to assess the relationship between parameters and clinical outcomes. Multivariate logistic regression analysis was used to evaluate the relationships between parameters and rapid renal progression. The model was adjusted for age, sex, the eGFR, the log-transformed UPCR, hypertension, CV disease, MBP, HbA1c, hemoglobin, albumin, BMI, log-transformed cholesterol, log-transformed CRP, and phosphorus, according to the literature and our previous publications^33, 34^.

We estimated the propensity scores for DM > 8 years, DR or positive diagnostic model using a non-parsimonious multivariable logistic regression model including all parameters shown in supplement table. The model was well-calibrated (Hosmer–Lemeshow test: P = 0.155) with reasonable discrimination (c statistic = 0.69). We matched patients between negative and positive groups with similar propensity scores to five, four, three, two and one decimal places in five repeated steps. In the first step, we multiplied the raw propensity scores by 100 000, then rounded it to the nearest value. This was repeated, multiplying by 10000, 1000, 100, and 10. A result of *P* < 0.05 was considered statistically significant. Statistical analysis was performed using the R 3.3.0 software (R Foundation for Statistical Computing, Vienna, Austria) and the Statistical Package for Social Sciences version 21.0 for Windows (SPSS Inc., Chicago, IL).

## Electronic supplementary material

Supplement Information
