# Supplementary material for: Diabetic Retinopathy and Clinical Parameters Favoring the Presence of Diabetic Nephropathy could Predict Renal Outcome in Patients with Diabetic Kidney Disease
Source: Sci Rep. 2017 Apr 21;7:1236. doi: 10.1038/s41598-017-01204-6 (PMC5430840; doi:10.1038/s41598-017-01204-6)
Supplement: Supplementary file 1 — Supplement Information [file 41598_2017_1204_MOESM1_ESM.pdf]

# Diabetic Retinopathy and Clinical Parameters Favoring the Presence of Diabetic Nephropathy could Predict Renal Outcome in Patients with Diabetic Kidney Disease

Chi-Chih Hung[1], Hugo You-Hsien Lin[1,2], Daw-Yang Hwang[1], I-Ching Kuo[1,2], Yi-Wen Chiu[1,3], Lee-Moay Lim[1], Shang-Jyh Hwang[1,3], Hung-Chen Chen[1,3]

[1]Division of Nephrology, Department of Internal Medicine, Kaohsiung Medical University Hospital, Kaohsiung Medical University.

[2]Department of Internal Medicine, Kaohsiung Municipal Ta-Tung Hospital, Kaohsiung Medical University.

[3]Faculty of Renal Care, College of Medicine, Kaohsiung Medical University, Kaohsiung, Taiwan.

[*]Corresponding author: Lee-Moay Lim

E-mail:limleemoay@yahoo.com.tw

Division of Nephrology, Department of Internal Medicine, Kaohsiung Medical University Hospital, Kaohsiung Medical University, No.100 Tzyou First Road, Kaohsiung 807, Taiwan.

TEL: +886-7-3121101ext7351

FAX: +886-7-3228721

## Support and financial disclosure declaration

The authors declare no conflicts of interest.

Supplement Table 1. Multivariate logistic regression for diabetic nephropathy in the biopsied patients with diabetic kidney disease.

| **variables** | **Odds ratio** | **95% CI** | ***P*-value** |
|---|---|---|---|
| Age (yr) | 1.005 | 0.982 to 1.029 | 0.698 |
| Male vs. female | 0.664 | 0.317 to 1.393 | 0.293 |
| eGFR (ml/min/1.73 m2) | 0.977 | 0.965 to 0.988 | 0.013 |
| Log-transformed UPCR | 1.112 | 0.569 to 2.179 | 0.793 |
| Diabetic retinopathy | 1.972 | 1.196 to 5.208 | 0.001 |
| Diabetes duration >5 year | 1.126 | 0.589 to 2.364 | 0.482 |
| HbA1c (%) | 1.449 | 1.205 to 1.745 | 0.005 |
| MBP (mmHg) | 0.995 | 0.983 to 1.009 | 0.253 |
| Hemoglobin (g/dl) | 1.032 | 0.967 to 1.100 | 0.365 |
| Albumin (g/dl) | 1.064 | 0.969 to 1.167 | 0.203 |
| Hematuria | 0.717 | 0.615 to 0.837 | 0.009 |

DM: diabetes mellitus, MBP: mean blood pressure, BMI: body mass index, eGFR: estimated glomerular filtration rate, UPCR: urine protein to creatinine ratio, CKD: chronic kidney disease, CRP: c-reactive protein, HbA1c: glycated hemoglobin.

Supplement Table 2. Characteristics and outcomes of propensity score-matched cohort by clinical parameters favoring diabetic nephropathy.

| | DM >8 years | | | Diabetic retinopathy | | | Diagnostic model | | |
|---|---|---|---|---|---|---|---|---|---|
| Variable | - | + | *P*-value | - | + | *P*-value | - | + | *P*-value |
| No. of patients | 296 | 296 | | 368 | 368 | | 308 | 308 | |
| **Demographics and comorbidity** | | | | | | | | | |
| Age (yr) | 63.1 (13.8) | 63.5 (13.2) | 0.714 | 65.0 (14.2) | 64.8 (12.2) | 0.843 | 64.4 (13.5) | 64.8 (12.4) | 0.736 |
| Sex (female) | 107 (36.1%) | 105 (35.5%) | 0.863 | 140 (38.0%) | 128 (34.8%) | 0.358 | 101 (32.8%) | 100 (32.5%) | 0.931 |
| Cardiovascular disease | 39 (13.2%) | 46 (15.5%) | 0.412 | 100 (27.2%) | 107 (29.1%) | 0.566 | 82 (26.6%) | 79 (25.6%) | 0.783 |
| Hypertension | 98 (33.1%) | 102 (34.5%) | 0.728 | 246 (66.8%) | 251 (68.2%) | 0.693 | 199 (64.6%) | 194 (63.0%) | 0.675 |
| Smoker | 42 (14.2%) | 43 (14.5%) | 0.906 | 47 (12.8%) | 59 (16.0%) | 0.207 | 48 (15.6%) | 51 (16.6%) | 0.742 |
| MBP (mmHg) | 99.4 (13.0) | 98.0 (12.3) | 0.163 | 99.6 (13.8) | 99.5 (13.7) | 0.973 | 98.2 (13.1) | 98.4 (12.7) | 0.901 |
| BMI (Kg/$m^2$) | 25.5 (4.2) | 25.2 (4.2) | 0.520 | 25.5 (4.4) | 25.4 (3.8) | 0.671 | 25.6 (4.3) | 25.5 (3.8) | 0.740 |
| **Laboratory data** | | | | | | | | | |
| eGFR (ml/min/1.73 $m^2$) | 37.6 (26.4-52.8) | 35.7 (27.2-48.6) | 0.227 | 34.3 (23.4-48.9) | 34.0 (25.6-47.5) | 0.672 | 36.3 (25.6-51.2) | 35.1 (27.3-46.2) | 0.471 |
| UPCR (mg/g) | 796 (278-2318) | 845 (286-2361) | 0.657 | 832 (284-2312) | 890 (290-2405) | 0.525 | 715 (250-2145) | 798 (280-2335) | 0.313 |
| Hemoglobin (g/dl) | 12.3 (2.1) | 12.4 (2.3) | 0.804 | 12.0 (2.1) | 12.2 (2.2) | 0.246 | 12.5 (1.7) | 12.3 (1.8) | 0.314 |
| Albumin (g/dl) | 3.9 (0.5) | 3.9 (0.5) | 0.889 | 3.9 (0.6) | 3.9 (0.6) | 0.759 | 3.9 (0.6) | 3.9 (0.6) | 0.275 |
| Total cholesterol (mg/dl) | 198 (170-226) | 198 (170-227) | 0.885 | 194 (165-225) | 191 (168-224) | 0.274 | 192 (169-225) | 192 (169-226) | 0.861 |
| Triglyceride (mg/dl) | 145 (105-212) | 139 (101-201) | 0.414 | 140 (100-202) | 145 (105-211) | 0.311 | 138 (98-199) | 138 (100-201) | 0.920 |
| Sodium (mEq/l) | 138.9 (3.3) | 138.4 (3.4) | 0.082 | 138.8 (3.4) | 138.5 (3.9) | 0.094 | 138.5 (3.1) | 138.1 (3.7) | 0.096 |
| Potassium (mEq/l) | 4.3 (0.5) | 4.3 (0.5) | 0.816 | 4.2 (0.5) | 4.3 (0.5) | 0.122 | 4.2 (0.5) | 4.3 (0.5) | 0.386 |
| Phosphorus (mg/dl) | 3.8 (0.8) | 3.8 (0.8) | 0.907 | 3.9 (0.9) | 3.9 (0.7) | 0.369 | 3.8 (0.8) | 3.9 (0.8) | 0.882 |
| Uric acid (mg/dl) | 7.4 (2.0) | 7.4 (1.8) | 0.729 | 7.6 (2.0) | 7.5 (1.8) | 0.235 | 7.9 (2.0) | 7.7 (1.8) | 0.153 |
| C-reactive protein (mg/dl) | 11.7 (28.8) | 11.7 (25.6) | 1.000 | 9.7 (25.9) | 9.5 (24.3) | 0.901 | 11.3 (29.2) | 9.5 (23.8) | 0.387 |
| Hba1c (%) | 7.8 (1.3) | 7.6 (2.0) | 0.273 | 7.1 (1.5) | 7.2 (1.3) | 0.547 | 7.1 (1.6) | 7.2 (1.4) | 0.241 |
| **Outcome** | | | | | | | | | |
| Rapid renal progression | 78 (27.9%) | 91 (31.0%) | 0.416 | 90 (25.1%) | 114 (31.7%) | 0.049 | 71 (23.6%) | 91 (30.1%) | 0.069 |
| ESRD | 24 (8.1%) | 27 (9.1%) | 0.664 | 34 (9.3%) | 49 (13.3%) | 0.080 | 20 (6.5%) | 31 (10.4%) | 0.011 |
| All-cause mortality | 43 (14.5%) | 36 (12.2%) | 0.397 | 46 (12.5%) | 54 (14.7%) | 0.389 | 37 (12.0%) | 38 (12.3%) | 0.901 |
| Cardiovascular events | 31 (10.5%) | 33 (11.1%) | 0.798 | 47 (12.8%) | 53 (14.4%) | 0.519 | 34 (11.0%) | 40 (13.0%) | 0.457 |

DM: diabetes mellitus, MBP: mean blood pressure, BMI: body mass index, eGFR: estimated glomerular filtration rate, UPCR: urine protein to creatinine ratio, HbA1c: glycated hemoglobin, ESRD: end-stage renal disease. Data are presented as the mean (standard error), median (interquartile range), or count (percentage).

Supplement Table 3. Associations between clinical parameters favoring diabetic nephropathy and clinical outcomes in propensity score-matched cohort.

| | DM >8 years | | | Diabetic retinopathy | | | Diagnostic model | | |
|---|---|---|---|---|---|---|---|---|---|
| | - | + | ***P*-value** | - | + | ***P*-value** | - | + | ***P*-value** |
| ***HR for ESRD*** | | | | | | | | | |
| Unadjusted | 1 (reference) | 0.98 (0.56-1.69) | 0.930 | 1 (reference) | 1.37 (0.88-2.13) | 0.159 | 1 (reference) | 1.76 (1.03-3.04)* | 0.040 |
| Fully-adjusted | 1 (reference) | 1.09 (0.60-1.98) | 0.766 | 1 (reference) | 1.80 (1.14-2.86)* | 0.012 | 1 (reference) | 1.89 (1.02-3.52)* | 0.045 |
| ***OR for rapid renal progression*** | | | | | | | | | |
| Unadjusted | 1 (reference) | 1.16 (0.81-1.66) | 0.416 | 1 (reference) | 1.39 (1.00-1.92) | 0.050 | 1 (reference) | 1.40 (0.97-2.01) | 0.070 |
| Fully-adjusted | 1 (reference) | 1.36 (0.90-2.04) | 0.142 | 1 (reference) | 1.56 (1.07-2.26)* | 0.020 | 1 (reference) | 1.34 (0.89-2.01) | 0.157 |
| ***HR for all-cause mortality*** | | | | | | | | | |
| Unadjusted | 1 (reference) | 0.77 (0.50-1.21) | 0.259 | 1 (reference) | 1.14 (0.77-1.70) | 0.502 | 1 (reference) | 0.95 (0.60-1.49) | 0.814 |
| Fully-adjusted | 1 (reference) | 0.86 (0.55-1.35) | 0.562 | 1 (reference) | 1.17 (0.77-1.76) | 0.461 | 1 (reference) | 0.78 (0.49-1.24) | 0.247 |
| ***HR for CV events*** | | | | | | | | | |
| Unadjusted | 1 (reference) | 1.00 (0.61-1.63) | 0.984 | 1 (reference) | 1.08 (0.73-1.61) | 0.698 | 1 (reference) | 1.11 (0.71-1.76) | 0.642 |
| Fully-adjusted | 1 (reference) | 1.17 (0.70-1.94) | 0.543 | 1 (reference) | 1.28 (0.85-1.92) | 0.231 | 1 (reference) | 1.04 (0.64-1.68) | 0.875 |

Model adjusts for age, sex, eGFR, log-transformed UPCR, HbA1c, hypertension, cardiovascular disease, MBP, albumin, hemoglobin, BMI, log-transformed CRP, BMI, log-transformed cholesterol, phosphate and ACEI/ARB.
